# Editorial Comment: Development and validation a task-specific checklist for a microsurgical varicocelectomy simulation model

**DOI:** 10.1590/S1677-5538.IBJU.2019.0571.1

**Published:** 2020-07-31

**Authors:** Rodrigo R. Vieiralves

**Affiliations:** 1 Hospital Federal da Lagoa Serviço de Urologia Rio de Janeiro RJ Brasil Serviço de Urologia, Hospital Federal da Lagoa, Rio de Janeiro, RJ, Brasil

## COMMENT

Microsurgery training is far from becoming a reality in the urologist practice. Due to the limitations of its application and aiming to increase the accessibility of this important tool, some experimental models have emerged over time (1, 2). In this very interesting article conducted at CEFET and Federal University of Minas Gerais, Brazil, a task-specific checklist of technical skills for microsurgical varicocelectomy was developed (3). As we know, a varicocele is an abnormal dilation of the pampiniform plexus of the testis. In up to 40% of infertile men, a palpable varicocele is found, while the prevalence of a varicocele in the general male population is about 15%. The benefit of varicocele repair must be weighed by the risk associated with the procedure, so it is important to select the procedure with the greatest success and lowest rate of complications. Microsurgical varicocelectomy, low inguinal or subinguinal (4), is preferred by many urologists and specialists in male infertility, as it is associated with a higher success rate, facilitating the identification of vascular structures and lymphatic vessels (5, 6). The surgery involves a complex number of factors for its true success. The preparation of the surgical field itself with the correct use of the microscope, the identification of vascular structures such as spermatic arteries and veins and also the lymphatic vessels with the correct handling of these structures are very important. In this context, this paper becomes naturally relevant, as it deals with a field that is still little explored in our specialty, with microsurgical ability still being an entry barrier to urological development in this scenario.

In this article with carefully elaborated methodology, a validated checklist was developed, allowing the assessment of surgical skills in the microsurgical treatment of varicocelectomy. Based on 4 requirements (handling the microscope, recognition of the fascia and identification of vessels, correct dissection and differentiation between arteries and veins, ligatures and the section of the vessels) was given a note regarding the performance in this technique, allowing to evaluate microsurgical abiliity in the treatment of varicocele.

However, some relevant aspects need to be highlighted. First we will refer to the simulation model used: The model used a human placenta that was prepared to simulate the spermatic cord. In addition to the difficulty in accessing placental tissue by the urological community, the consistency of this reconstructed funicle through a placental incision with allantoic membrane coating is unlikely to simulate the consistency of the external spermatic fascia. In the article, we did not identify the exact region of the placenta that should be chosen. It should include a placental artery but without reporting if it should be more proximal or distal to the umbilical cord. We understand that, according to the region of the placenta, the vascular net can in theory present different caliber, impacting on a non-realistic model. The vessels were also perfused with a dye solution (red for artery and blue for vein) which, in theory, would facilitate the diagnosis of an arterial or venous vascular structure. With regard to the development of the check list, it is noteworthy that only 14 urologists had previous practice in microvaricocelectomy and with a small experience (10 procedures / year). As they were recruited as judges in the elaboration of the questionnaire, we consider the “n” not high enough and it would be possible that aspects relevant to the procedure were left out. Also, during the construction of the validation, the presence of only two educational experts can also compromise the validation. Ideally, the agreement of a third expert could have been included in the experiment, increasing the reliability. Still, nothing about statistical tests for validation or reliability analysis was mentioned. Another point that should be mentioned is the low number of participants in the model and the simulation with only nine residents of the same service. We understand that results based on training of only 9 residents can present an important bias, due to individual aspects besides the fact that they are part of the same Hospital (similar previous training), making the external validation of this model difficult.

To conclude, we see in this important paper the presentation of task-specific check list for a microsurgical varicocelectomy simulation model, that is reproducible and quickly applicable. It allows assessment of surgical skills and thus offers an evaluation method of the stage of progression in the microsurgical varicocelectomy training process. Being an unprecedented model, we understand that it can serve as a basis for new checklists and realistic simulation models in the scenario of microsurgery applied to urology.
